# High-performance biocomputing for simulating the spread of contagion over large contact networks

**DOI:** 10.1186/1471-2164-13-S2-S3

**Published:** 2012-04-12

**Authors:** Keith R Bisset, Ashwin M Aji, Madhav V Marathe, Wu-chun Feng

**Affiliations:** 1 Virginia Bioinformatics Institute, Virginia Tech, Blacksburg, Virginia, USA; 2 Department of Computer Science, Virginia Tech, Blacksburg, Virginia, USA; 3 Department of Electrical & Computer Engineering, Virginia Tech, Blacksburg, Virginia, USA

## Abstract

**Background:**

Many important biological problems can be modeled as contagion diffusion processes over interaction networks. This article shows how the EpiSimdemics interaction-based simulation system can be applied to the general contagion diffusion problem. Two specific problems, computational epidemiology and human immune system modeling, are given as examples. We then show how the graphics processing unit (GPU) within each compute node of a cluster can effectively be used to speed-up the execution of these types of problems.

**Results:**

We show that a single GPU can accelerate the EpiSimdemics computation kernel by a factor of 6 and the entire application by a factor of 3.3, compared to the execution time on a single core. When 8 CPU cores and 2 GPU devices are utilized, the speed-up of the computational kernel increases to 9.5. When combined with effective techniques for inter-node communication, excellent scalability can be achieved without significant loss of accuracy in the results.

**Conclusions:**

We show that interaction-based simulation systems can be used to model disparate and highly relevant problems in biology. We also show that offloading some of the work to GPUs in distributed interaction-based simulations can be an effective way to achieve increased intra-node efficiency.

## Introduction

Network-based models are natural generalizations of stochastic mass action models to stochastic dynamics on arbitrary interaction networks. Consider, for example, a typical network model for computational epidemiology:

• a network with labeled vertices and edges, representing people and possible disease transmission paths, respectively;

• vertex labels, representing the corresponding person's state of health, i.e., susceptible (S), infectious (I), or recovered (R);

• edge labels, representing the probability of transmission from one person to another; and

• discrete-time dynamics, corresponding to percolation across the graph, i.e., the label on a vertex in state S changes to I based on independent Bernoulli trials for each neighbor in the state I with the probability specified by the edge between them; the label on a vertex in state I changes to R with some fixed probability.

We have developed synthetic networks that can be used to model diffusion processes for large numbers of agents (10^6 ^- 10^9^) in a variety of domains [1]. These regional (and national scale) networks were originally created to model the spread of infectious diseases through large human populations [2]. They have since been adapted to also model the spread of malware in wireless networks [3], and the spread of information, fads, and norms through friendship networks. Recently, we have also extended our methods to develop synthetic human cellular networks that are useful for modeling the human immune responses to two gastroenteric pathogens - these networks require simulating up to 10^9 ^individual cells [4]. We have successfully computed the spread of influenza on a network representing the population of the United States (270 million people performing 1.4 billion activities) on a CPU cluster with 768 cores.

Today, computational performance improvements are increasingly achieved through parallelism within a chip, both in traditional multi-core architectures as well as the many-core architectures of the graphics processing unit (GPU). Amongst the most prominent many-core architectures are the GPUs from NVIDIA and AMD/ATI, which can accelerate a large variety of scientific applications, at very affordable prices [5-7]. Our previous work on computational efficiency focused on reducing the communication overhead and improving the inter-node performance of our simulation systems [8]. The work described in this paper focuses on improving the intra-node performance through the use of GPU accelerators.

Our agent-based models are built on rigorous mathematical foundations - this is one of the unique aspects of our work. These are called Graphical Discrete Dynamical Systems [1,9-11] In the beginning, we used the term sequential dynamical systems to describe these mathematical objects. We now use the term graphical dynamical systems to be consistent with other graphical models that have been proposed in the literature, e.g. graphical inference, graphical games, etc. The mathematical model consists of two parts: (i) a co-evolving graphical discrete dynamical system framework (CGDDS) that captures the co-evolution of system dynamics, interaction network and individual agent behavior, and (ii) a partially observable Markov decision process that captures various control and optimization problems formulated on the phase space of this dynamical system. Informally speaking, a CGDDS consists of the following components: a dynamic graph *G_t_*(*V_t_*, *E_t_*) in which vertices represents individual objects (agents) and edges represent a causal relationship that is usually local, a set of local state machines (automata), one for each vertex specifying the behavior of the agents and a set of update functions, one per edge that describes how an edge can be modified as a function of the state of its endpoints. The state and behavioral changes of individual objects are a function of their internal state and its interaction with neighboring agents. These neighbors change over time and space, and thus the model needs to explicitly represent this network evolution.

CGDDSs serve as a bridge between mathematical simulation theory and HPC design and implementation. Like state charts and other formal models, they can be used for formal specification, design and analysis of multi-scale biological and social systems. CGDDSs allow us to reason about efficient implementation and specification of interactive simulations. Second, the aggregate behavior of iterated compositions of local maps that comprise a CGDDS can be understood as a (specific) simulated algorithm together with its associated and inherent computational complexity. We have called this the algorithmic semantics of a CGDDS (equivalently, the algorithmic semantics of a dynamical system or a simulation). It is particularly important to view a composed dynamical system as computing a specifiable algorithm with provable time and space performance. Together, the formal framework allows us to understand how and when can network based models be mapped efficiently onto parallel architectures.

### Example modeling domains

While originally created for modeling infectious diseases, EpiSimdemics has also been used to model the spread of malware in wireless networks, the spread of obesity and smoking in friendship networks, and inflammation in humans. Below, we describe one of these domains, Computational Epidemiology, in greater detail.

The spread of infectious diseases such as H1N1, or recent outbreaks of Cholera in Haiti are important public health problems facing the global community. Computational models of disease spread in a population can support public health officials by helping them to assess the scope and magnitude of the spread and evaluate various methods for containing the epidemic. Models of disease propagation over realistic networks for planning, response and situation assessment have been used during the recent epidemic outbreaks [12-15]. A first step in developing such models is the synthesis of realistic social contact networks.

Following Beckman et al. [10,14,16-18], we estimate a contact network in four steps: (1) population synthesis, in which a synthetic representation of each household in a region is created; (2) activity assignment, in which each synthetic person in a household is assigned a set of activities to perform during the day, along with the times when the activities begin and end; (3) location choice, in which an appropriate real location is chosen for each activity for every synthetic person; and (4) contact estimation, in which each synthetic person is deemed to have made contact with a subset of other synthetic people.

The contact network is a graph whose vertices are synthetic people, labeled by their demographics, and whose edges represent contacts determined in step four, labeled by conditional probability of disease transmission.

Each of the four steps makes use of multiple stochastic models; the first three incorporate observed data for a particular region. The synthetic population is generated using an iterative proportional fit to joint distributions of demographics taken from a census [19]. It consists of a list of demographic variables such as age, gender, income, household size, and education. Activities like school, work, and shopping are assigned using a decision tree model based on household demographics fit to activity or time-use survey data. This step creates a "personal day planner" for each person in the synthetic population. Activity locations are chosen based on a gravity model and land use data. That is, every synthetic activity produced in step three is assigned to an actual location - office building, school, shopping mall, etc. - based on its distance from the person's previous activity and its attractiveness, a measure of how likely that the activity happens there.

Population synthesis uses a non-parametric, data-driven model based on US census data for the year 2000 (currently being updated to the 2010 census), including the Public Use Microdata Sample [20]. The actual activity templates assigned to households are data-driven, based on the National Household Transportation Survey [21]. The gravity model used for location choice contains nine parameters. The locations' addresses and attractor values are derived from Dun & Bradstreet's Strategic Database Marketing Records.

Disease propagation is modeled by

(1) $$p_{i\to j}=1-{\left(1-r_{i}s_{j}\rho \right)}^{\tau }$$

where *p_i_*_→_*_j _*is the probability infectious individual *i *infecting susceptible individual *j*, *τ *is the duration of exposure, *r_i _*is the infectivity of *i*, *s_j _*is the susceptibility of *j*, and *ρ *is the transmissibility, a disease specific property defined as the probability of a single completely susceptible person being infected by a single completely infectious person during one minute of exposure [1].

### The EpiSimdemics simulator

EpiSimdemics [2,8] is an interaction-based, high-performance computing modeling environment to study problems represented as a CGDDS. EpiSimdemics allows us to study the joint evolution of contagion dynamics, interactor (i.e., agent) behavior and interaction networks as a contagion spreads. The interaction network is represented as a bi-partite graph, with interactors (e.g., people or cells) and locations (e.g., building or tissue types) represented as nodes and visits of locations by interactors represented as edges. Each interactor's state is represented as one or more Probabilistic Timed Transition Systems (PTTS). A PTTS is a finite state machine where transitions can be timed and probabilistic.

**Figure 1:**
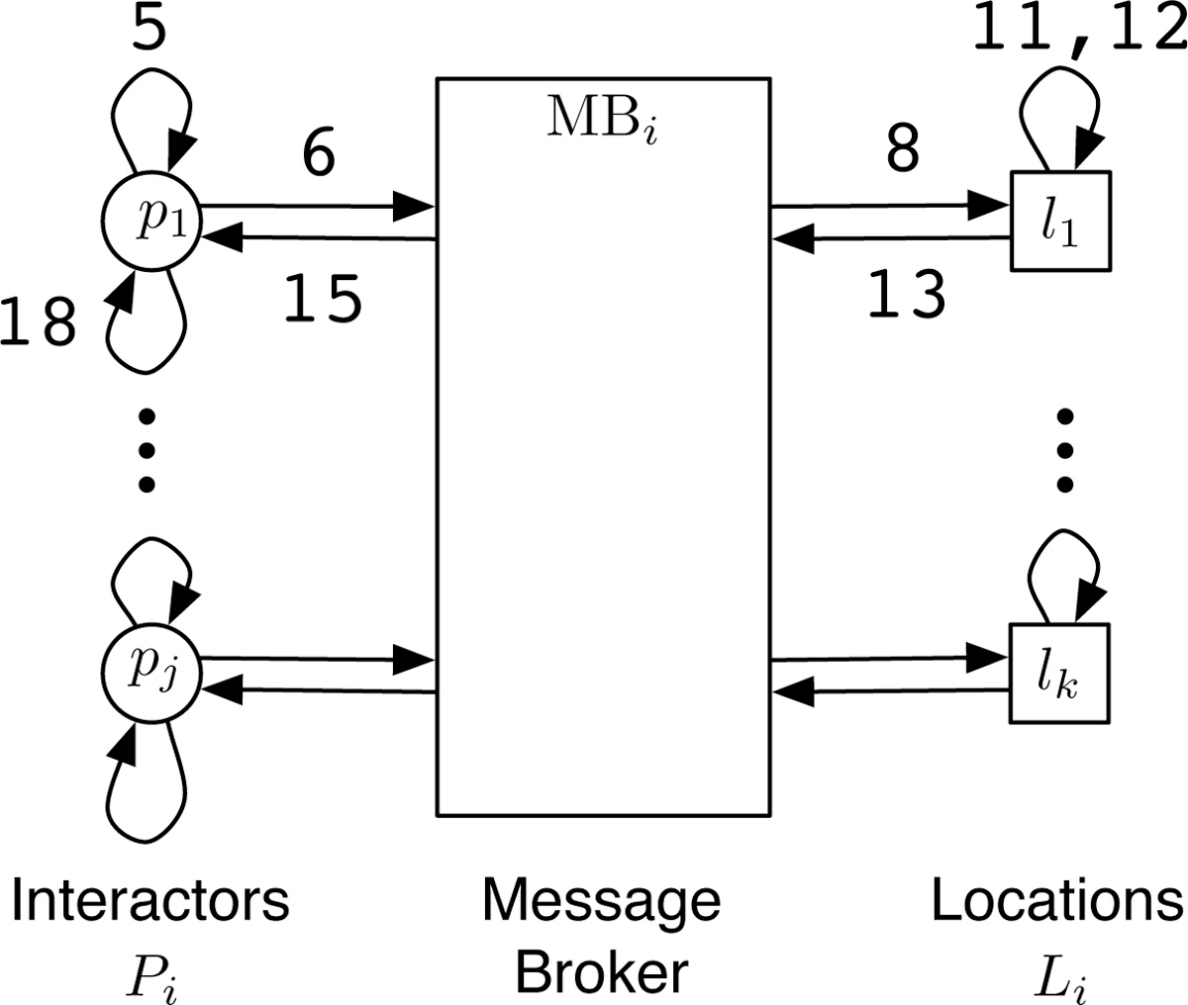
**The computational structure of EpiSimdemics**. The numbers in the diagram correspond to line numbers in the algorithm in Figure 2.

**Figure 2:**
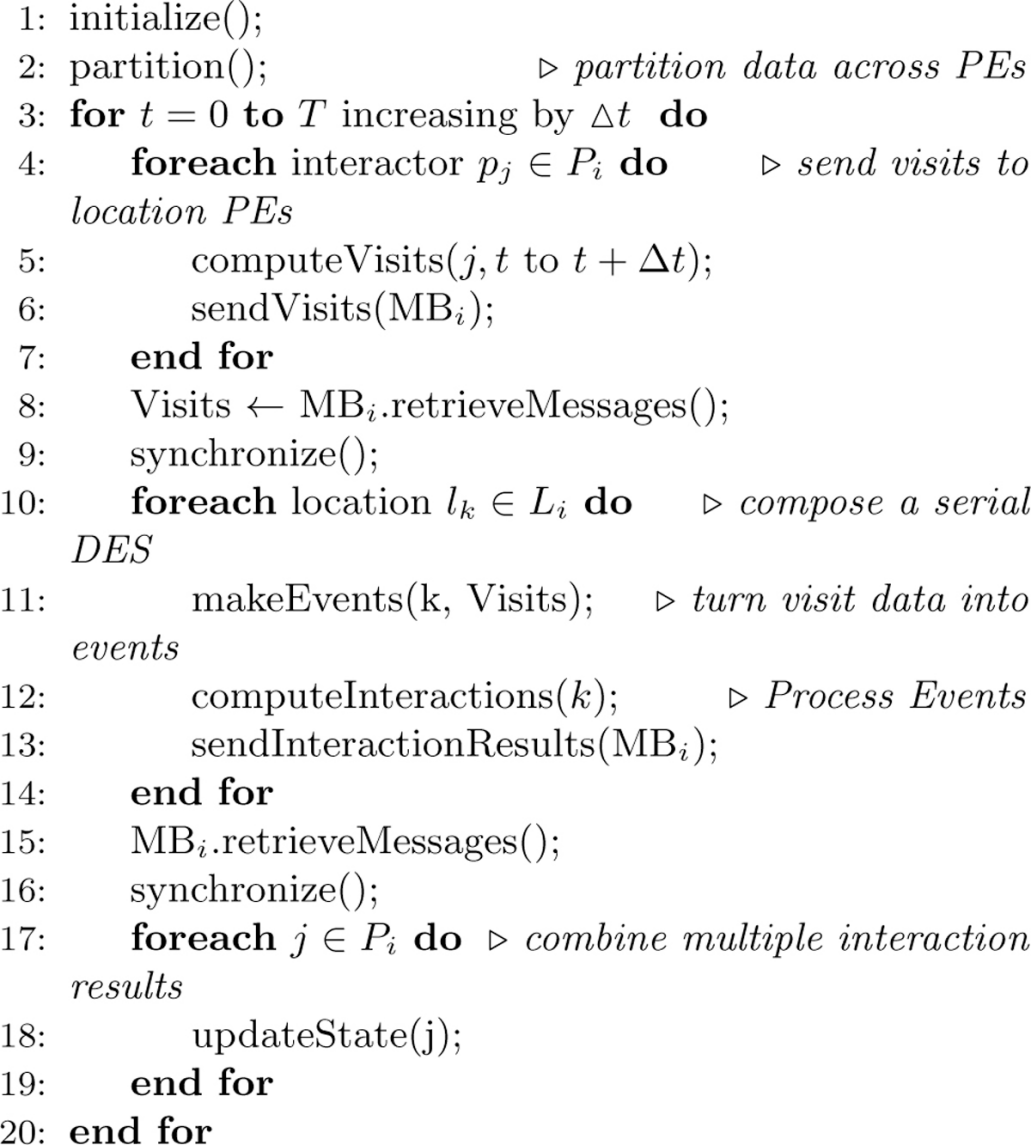
**Parallel algorithm of EpiSimdemics**. The line numbers in the algorithm correspond to numbers in Figure 1.

The computation structure of this implementation, shown in Figure 1, consists of three main components: interactors, locations and message brokers. Given a parallel system with *N *cores, or Processing Elements (PEs), interactors and locations are first partitioned in a round-robin fashion into *N *groups denoted by *P*_1_, *P*_2_,..., *P_N _*and *L*_1_, *L*_2_,..., *L_N_*, respectively. Each PE then executes all the remaining steps of the EpiSimdemics algorithm, given in Figure 2, on its local data set (*P_i_*, *L_i_*). Each PE also creates a copy of the message broker, *MB_i_*. Next, a set of *visit messages *are computed for each interactor *P_i _*for the current iteration, which are then sent to each location (which may be on a different PE) via the local message broker. Each location, upon receiving the visit messages, computes the probability of spread of contagion for each interactor at that location. Outcomes of these computations, called interaction results, are then sent back to the "home" PEs of each interactor via the local message broker. Finally, the interaction result messages for each interactor on a PE are processed and the resulting state of each affected interactor is updated. All of the PEs in the system are synchronized after each iteration, so that the necessary data is guaranteed to have been received by the respective PEs. The above steps are executed for the required number of iterations (e.g., days in an epidemiology simulation), and the resulting interaction network dynamics are analyzed in detail. The computation that takes place on line 12 of the algorithm consumes roughly 60% of the execution time of the system. It is this portion of the computation that is offloaded to the GPU. Note that this section of the computation is purely serial in that it does not require any inter-process communication. Therefore, any speedup that is gained through this offload will be applied linearly across the entire distributed computation.

In general, there is a trade-off between computational generality and computational efficiency. The more tightly constrained the problem being simulated, the more the semantics of the problem can be exploited to increase computational efficiency. EpiSimdemics is designed to compute diffusion processes in parallel across a wide range of domains. For this reason, efforts at increasing the computational efficiency of the simulator are targeted at high-level algorithmic and implementation features that do not, in general, depend on the semantics of the particular diffusion process being studied. Any diffusion process that contains an inherent latent period such that the results of an interaction between two agents does not immediately effect other interactions should be able to be simulated efficiently. For instance, when a person becomes infected through an interaction with a contagious person, the newly infected person is not immediately contagious themselves.

### The NVIDIA graphics processing unit (GPU) architecture

The NVIDIA GPU (or *device*) consists of a set of streaming multiprocessors (SMs), where each SM consists of a group of scalar processor (SP) cores with a multi-threaded instruction unit. The actual number of SMs vary depending on the different GPU models. The NVIDIA Tesla S2050, which we used for our experiments, consists of 14 SMs, each with 32 SP cores, making a total of 448 SP cores.

All the SMs on the GPU can simultaneously access the *device memory*, which can be upto 6 GB in capacity. The device memory can also be read or written to by the host CPU processor. Each SM has faster on-chip *shared memory*, which can be accessed by all the SPs within the SM and are up to 48 KB in size. The shared memory can be considered to be a data cache that is explicitly managed by the computation executing on the GPU.

#### The CUDA programming model

CUDA (Compute Unified Device Architecture) [22] is the parallel programming model and software environment provided by NVIDIA to run applications on their GPUs, programmed via simple extensions to the C programming language. CUDA follows a code offloading model, i.e., compute-intensive portions of applications running on the host CPU processor are typically offloaded onto the GPU device for acceleration. The *kernel *is the portion of the program that is compiled to the instruction set of the GPU device and then offloaded to the device before execution. For this paper, we have used CUDA v3.1 as our programming interface to the GPU.

Each kernel executes as a group of threads, which are logically organized in the hierarchical form of grids and blocks of threads. The dimensions of the blocks and the grid can be specified as parameters before each kernel launch. The kernel is mapped onto the device such that each thread block is executed on only one SM, and a single SM can execute multiple such thread blocks simultaneously, depending on the memory requirements of each block. The on-chip shared memory of an SM can be accessed only by the thread block that is running on the SM, while the global device memory is shared across all the thread blocks in the kernel.

## Results and discussion

### Algorithm - EpiSimdemics on a GPU

As discussed before, the most computationally intensive part of the application, i.e., *Compute-Interactions *should be *offloaded *to the GPU for faster execution. Since each compute node in our test cluster has a GPU attached to it, every node accelerates the *Compute-Interactions *phase of the algorithm on the dedicated GPU device, while performing the rest of the computations on the CPU. Moreover, since each node in the cluster has identical computation and communication patterns, we can investigate the intra-node parallelization methods for a single GPU device in isolation. More specifically, the GPU offload process can be broken down into the following steps:

1. Transfer the input data from the CPU's main host memory to the GPU's device memory.

2. Invoke the GPU kernel method (*computeInteractions()*), which does the parallel computation on the GPU device.

3. Transfer the output data from the GPU's device memory back to the CPU's main host memory.

We invoke the CUDA kernel once per simulation iteration. Inside each kernel, we loop over all the set of locations that are being processed by the current node, and calculate the contagion spread information for each location for a simulation iteration. The interactions between agents are computed between all pairs of co-located occupants, where the interaction is modeled, for example, by Equation 1. For GPU-EpiSimdemics, the locations, the interactors, and their respective PTTS states are transferred to the GPU's device memory as input. After processing, the interaction result messages are transferred back to the CPU's main memory, which are then communicated to the other nodes in the cluster, as necessary.

#### Semantic approximation

In the CPU-only implementation of EpiSimdemics, interactions between agents are computed using a sequential discrete event simulation (DES). Each visit by an interactor to a location is converted into a pair of events, one each for arrivals and departures. The events for each agent that visits a location during an iteration is placed in a list sorted by event arrival or departure time, as appropriate. The events in the list are then processed, starting with the event with the earliest time. An arrive event causes the agent to be added to the occupant list for the location. A depart event first removes the agent from the occupant list, and then computes the interactions, pair-wise, between the departing agent and each agent remaining in the occupancy list.

The initial GPU version of this algorithm was a straightforward implementation of the CPU algorithm. It did not scale well, taking about the same time on the GPU as on the CPU. This has been reported in the literature for other similar applications [23]. There are two main reasons for this. First, the amount of data that needed to be transferred from the CPU to the GPU was large compared to the amount of computation that needed to be performed. Second, the GPU's SMs perform best when executing in a single-instruction multiple-data (SIMD) fashion, which is hard to achieve in a DES.

In order to improve performance, the DES-based algorithm was converted to a time-stepped algorithm, following a similar procedure to that used in [24]. The iteration was divided up into time bins, and a matrix created for each location indicating, for each agent and bin, how long the agent was present in that location during the time covered by that time bin. The length of the interaction between two agents within a time bin is the minimum of their entries in the matrix. A time bin of five minutes was used for the results presented in this paper, which gives a good performance while maintaining accuracy, as shown in the section on Program Correctness.

The time-stepped algorithm approximates the DES algorithm. It may overestimate the interaction time and it is possible for two agents to interact even if they do not visit a location at the same time, but are placed in the same time bin (i.e., one leaves shortly before the other arrives). An overestimation of the contact time between two agents can occur only in, at most, two time bins: the one containing the maximum of the arrival times and one containing the minimum of the departure times. In other words, the first and last bins in which the agents are both at the location. These may be the same bin. In all time bins between these two, both agents are at the location for the time covered by the time bin, and no over-estimation can occur. Therefore, the maximum error for a single contact is twice the bin size. Reducing the size of the time bins reduces the error in the approximation, at the expense of transferring more data to the GPU.

As an example, consider four agents, A1-A4, visiting a location, with the arrival and departure times given in Table 1. The iteration is divided into three 60 minute time bins: B1 covers time 0-60, B2 covers 60-120, and B3 covers 120-180. The length of occupancy of each agent in each of the time bins is also shown in Table 1. The approximated length of interaction for each pair of agents for each time bin is shown in Table 2, along with the total approximated interaction time and the actual interaction time. In particular, note the interaction time between A1 and A2, which is approximated by the time-stepped algorithm as 10 minutes, when they are never in the location at the same time.

**Table 1:** Example of approximation algorithm.

Agent	Arrive	Depart	B1	B2	B3
A1	30	70	30	10	0
A2	90	150	0	30	30
A3	55	130	5	60	10
A4	65	95	0	30	0

**Table 2:** Results of approximation algorithm for each pair of interactions, and actual interaction time.

Interaction	B1	B2	B3	Approx	Actual
A1↔A2	0	10	0	10	0
A1↔A3	5	10	0	15	15
A1↔A4	0	10	0	10	5
A2↔A3	0	30	10	40	40
A2↔A4	0	30	0	30	5
A3↔A4	0	30	0	30	30

### GPU implementation

In this section, we discuss the mapping of the *Compute-Interactions *phase to the computational and memory units within a GPU. Then, we describe how we used all the available CPU and GPU resources efficiently to gain more speed-up and scalability.

#### Mapping to the computational units

**Figure 3:**
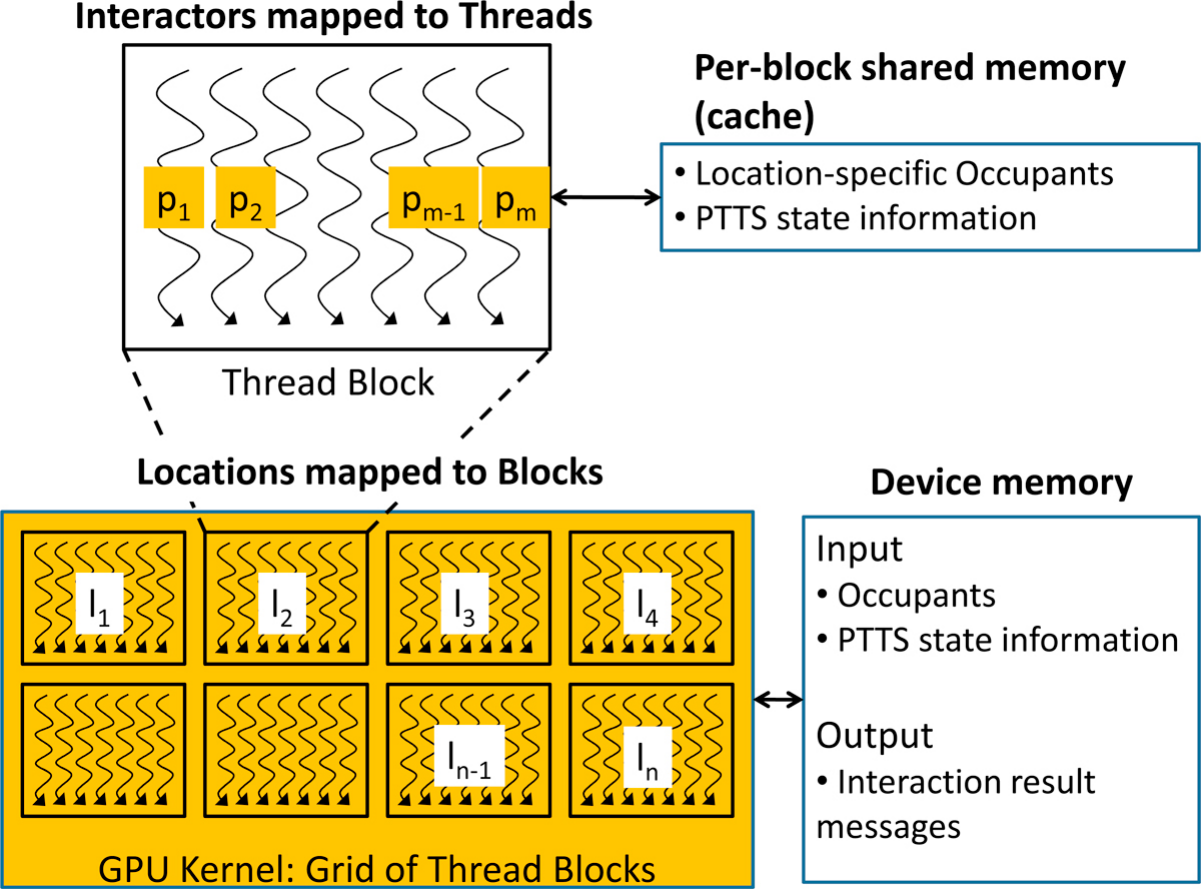
**Mapping of Compute-Interactions to the NVIDIA GPUs**. *l*_1 _-*l_n _*denote the set of independent locations, and *p*_1_-*p_m _*indicate the set of interactors within one of the locations.

The threads in the GPU kernel are grouped as blocks in a grid, and parallelism can be exploited at two layers, i.e., independent tasks can be assigned to different *blocks*, while the *threads *in each block can work together to solve the assigned task. We can see that the locations can be processed in parallel, and so the *Compute-Interactions *phase of EpiSimdemics can map very well to the hierarchical CUDA kernel architecture, as shown in Figure 3. More specifically, the set of independent locations can be assigned to different blocks for parallel processing. The interactors within a location are then assigned to independent threads within the block. The threads (occupants) can cooperatively work together and generate the set of interaction result messages for their location.

#### Mapping to the memory units

All the blocks in a CUDA kernel can access the entire device memory of the GPU. The input data is therefore transferred to the device memory from the CPU. The input data contains information about the occupants in all the locations, and the PTTS state information of each occupant in every location. However, access to the device memory is very slow (400-600 clock cycles [22]) when compared to the faster shared memory or cache memory. Moreover, the faster shared memory is local to each CUDA block, where each block processes a single location. We make use of the faster shared memory by first explicitly copying the relevant occupants and PTTS state information from the device memory to the caches, and then do the processing. The movement of data from the device's global memory to the faster shared memory is slow, but it needs to be done just once per location. The interaction result messages (output data) are directly stored in the device's global memory. Figure 3 shows the mapping of the input data to the different memory hierarchies in the GPU.

We further optimized the device memory accesses and data access patterns in the shared memory, as suggested in the CUDA programming guide [22].

#### CPU-GPU co-scheduling

The GPU kernel offload method uses the available intra-node parallelism in a GPU and improves the overall scalability of EpiSimdemics. However, this requires each CPU to have a dedicated GPU device to which the tasks can be offloaded for acceleration. But, each node in any present day cluster will usually have more CPUs (2 - 32) than the number of GPUs (1 - 4). For example, our test cluster is made up of 8 nodes with 32 CPU cores per node. Each node is attached to a dual-GPU system, which means that 32 CPU cores in every node are contending with each other to offload the kernels to 2 GPUs. The scalability of EpiSimdemics will be poor if the kernels are offloaded in the default way (by blocking), because CPU resources will be wasted in just waiting for a free GPU. Next, we present our ongoing work, where we have explored different kernel offload techniques to improve the scalability of general multi-CPU-multi-GPU applications [25]. For our discussions we assume that each node contains *c *CPU cores, and each node is attached to *g *GPU devices.

##### Default offload

In the default offload method, either *g *CPU cores in a node offload the complete kernel to the *g *GPU's (one host core per GPU), or all the c CPU cores offload a fraction of the kernel simultaneously to the *g *GPU's (*c*/*g *hosts per GPU). We should point out that the multi-CPU offload approach results in the queuing of both GPU data transfers and kernel executions, since concurrent accesses to the GPU's from multiple GPU contexts is not yet supported by NVIDIA. Also, the order in which the CPU's access the GPU's is not defined and depends on the GPU's run-time system.

If the data and the kernel tasks scale according to the available processing elements, then both the above approaches will result in the same overall execution times. This is because the data transfers and kernel tasks can be simply aggregated and launched from the minimum set of CPU cores or split into all available CPU cores. In some applications, the amount of data transferred to or from the GPU will be constant, and does not depend on the kernel size (e.g., some graph analysis kernels require the complete graph in the device memory for different sized loads). For such cases, it is obviously better to aggregate the tasks into *g *GPU kernels, and offload them independently from *g *set of CPU cores. In effect, either types of applications can be marshaled such that, offloading either from *g *or *c *CPU cores produce similar effects.

##### Cooperative offload

While the default offload method has performance advantages over the CPU-only solutions for certain system configurations, it suffers from CPU slack, i.e., computational resources will be wasted in simply waiting for a free GPU. On the other hand, the cooperative offloading method removes the processor slack and keeps all the processing cores busy during the entire kernel execution.

In the cooperative offload method, the tasks to be executed on the CPU cores are distributed intelligently between CPU and GPU cores depending on the relative speedup of the GPU when compared to the CPU. Similar to the default offload method, there are a couple of ways to do the cooperative offloading - (1) Exclusive mode: *g *CPU cores are exclusively assigned to specific host cores to perform the GPU kernel offload, while the other *c *− *g *CPU cores execute the remaining CPU tasks, or (2) Round-robin mode: access to the GPU is distributed in a round-robin fashion across the different host cores in the node, and every CPU core performs useful computations before and after its turn to execute on the GPU. The CPU cores communicate the order of GPU execution via shared memory or explicit message passing protocols. Typically the threads or processes within the node asynchronously wait for a *ready *message from the immediately lower ranked thread/process, before proceeding to use the GPU. Similar to default offloading, the exclusive mode is more useful if the data transfer and kernel tasks can be aggregated to be launched from the minimum set of host CPU cores. However, some multi-threaded applications are not flexible in dynamic task distribution, and their kernels cannot be merged and divided as needed. Such applications will have to launch kernels from their individual threads, and can adopt the round-robin offloading method to avoid the GPU resource contention.

### Testing

#### Experimental setup

##### Data

To explore the performance and scalability of the GPU kernel, we chose a data set that fits in both the CPU and GPU memories within a single node. Our data set was synthetic population from the state of Delaware, consisting of 247, 876 persons and 204, 995 locations. All results are based on running EpiSimdemics for 55 simulated days and computing the average single day time across those 55 days.

##### Hardware

We ran our experiments on an eight-node cluster, where each node had four eight-core AMD processors. Each node was attached to two NVIDIA Tesla S2050 GPUs, which belong to the latest Fermi GPU architecture family. For most experiments, we used one CPU core and one GPU on a single node. To study our ongoing work of the different GPU offload methodologies, we use 8 CPU cores and both GPUs.

#### Execution profile

There are two measures of speedup that can be considered. The first is the speedup of a computational kernel offloaded to a GPU, as compared to execution only on a CPU. This measure also includes data structure setup done on the CPU, and data transfer time between the CPU and GPU (lines 11 and 12 in Figure 1). We refer to this as *kernel speedup*. The second measure compares the execution of the entire algorithm (lines 3 through 20), including the parts that were not considered for GPU offload. We refer to this as *application speedup*.

**Figure 4:**
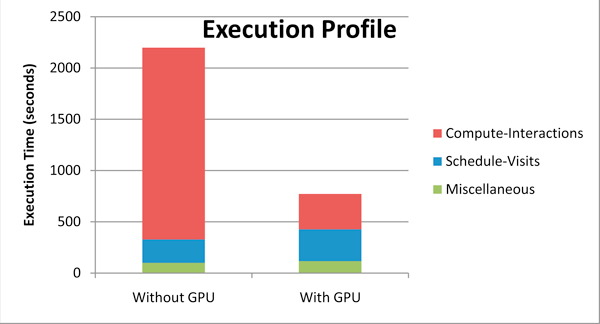
**Execution time of different steps in the EpiSimdemics algorithm**.

Figure 4 shows the distribution of the different steps of the EpiSimdemics algorithm, for both the CPU and GPU implementations. This result, in particular, was obtained by running the program on a single CPU and offloading the *Compute-Interactions *computation to one GPU. However, the distribution in the execution profile remains nearly the same when run on multiple cluster nodes. This results in a kernel speedup of six and an application speedup of 3.3 on a single compute node.

#### Scalability analysis

**Figure 5:**
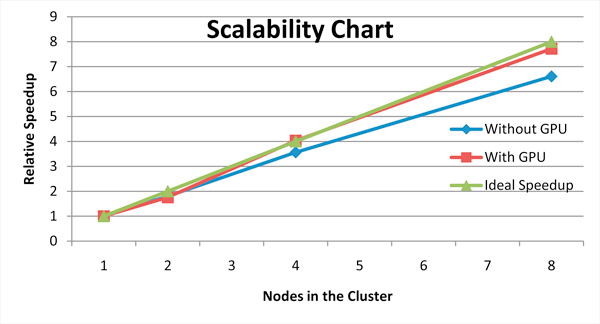
**Relative performance of EpiSimdemics with and without the GPU with respect to the ideal speedup**.

In this section, we will show the results of executing EpiSimdemics on up to eight nodes in the cluster. Ideally, in the CPU-only case, the performance improvement of a program should be proportional to the number of nodes in the system. In practice, we do not get perfectly linear scaling when we increase the number of nodes, because of the overhead incurred by communicating data among the different nodes. By offloading some of the computation within the node to a GPU, we can achieve super-linear speedup. Figure 5 shows the performance improvement of EpiSimdemics (with and without the GPU) relative to a single node system without a GPU. These results were obtained using only a single core and single GPU on each node. If multiple cores and GPUs are used, contention for the GPUs becomes an issue. This is handled through *cooperative offload*, discussed below.

#### Confirmation of program correctness

**Figure 6:**
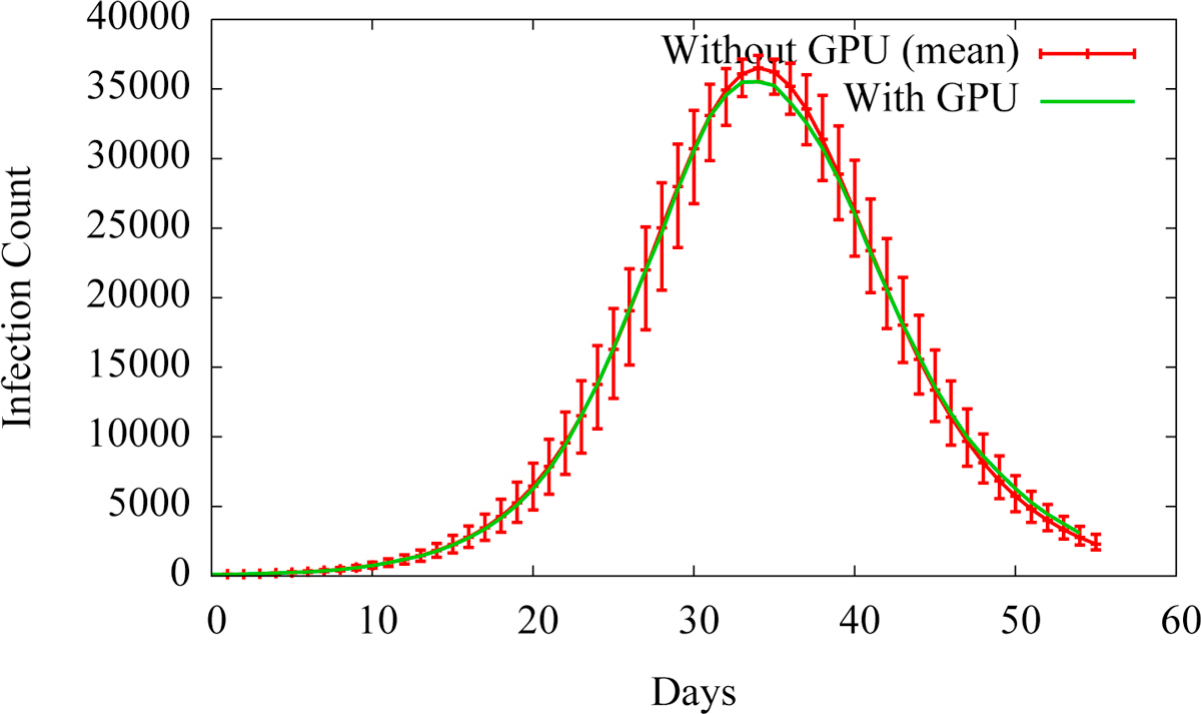
**Epi-curves validating the correctness of GPU-EpiSimdemics**.

We confirm the correctness of the contagion spread calculations when computed on the GPU by comparing results of the original CPU-only computation to the results of the GPU accelerated computation. We do this by plotting the epi-curves (i.e., new infections per day) that are generated by EpiSimdemics for both the CPU and GPU platforms as shown in Figure 6. The epi-curve for the CPU case is the mean of 25 runs, with error bars showing the variance within those runs. The epi-curve for the GPU case falls within the errorbars for the CPU case, thereby confirming that GPU-EpiSimdemics produces the same dynamics as the original EpiSimdemics.

#### Example disease spread

**Figure 7:**
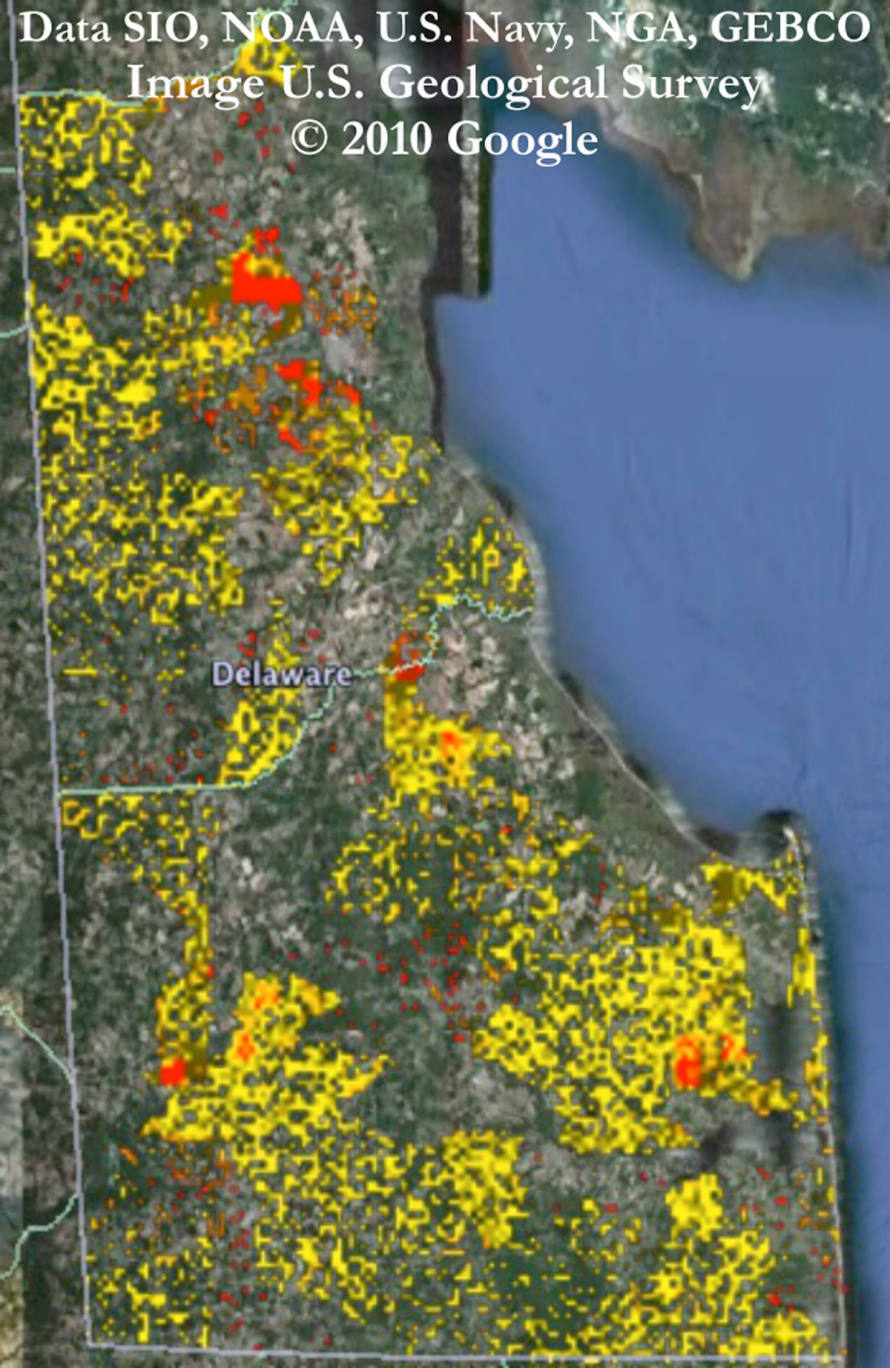
**Simulated disease spread on day 34 in Kent and Sussex counties in Delaware**. Yellow through red areas indicate increasing disease prevalence.

Figure 7 shows a snapshot of the spread of disease in Delaware's Kent and Sussex counties on day 34 of one simulation. The area of interest is divided into small blocks, and each block is colored according to the number of infected individuals who live in that block. Yellow blocks indicate areas of low disease prevalence, while red blocks indicate higher prevalence.

#### Evaluating the GPU offload methods

**Figure 8:**
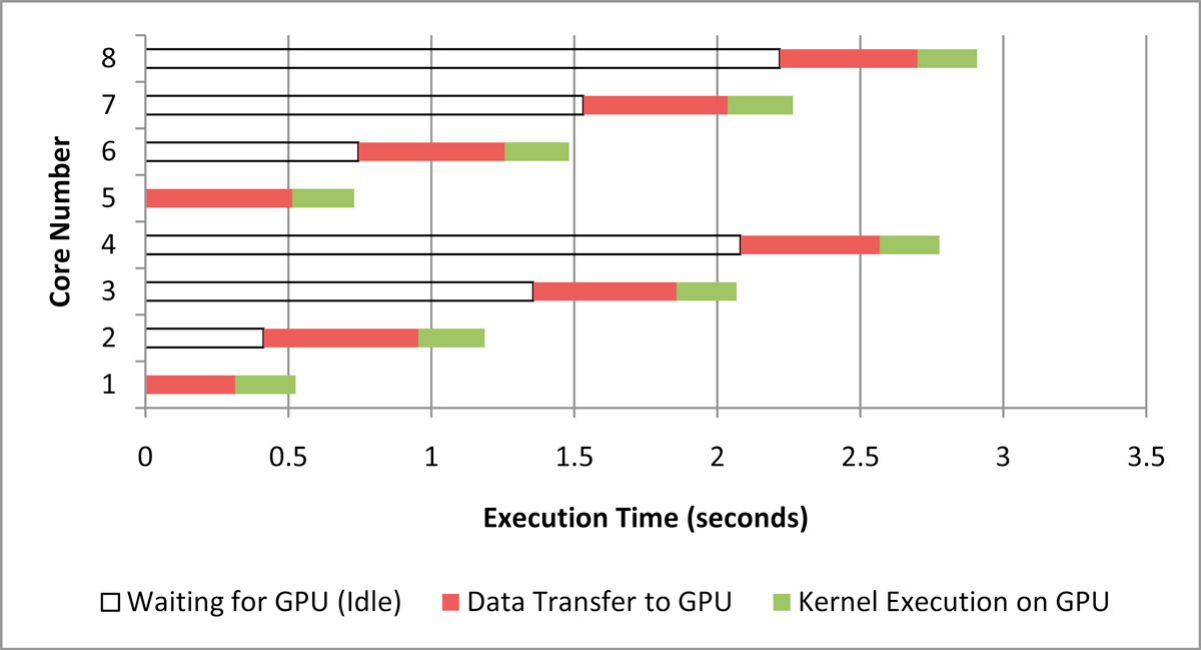
**Default kernel offload profile**.

Figure 8 shows the execution profile of all the 8 CPU cores while applying the default offload method, where the entire eligible computation is offloaded to the GPU, and each core must wait for use of the GPU. The execution time in this and the next two figures is the average compute time across all 55 iterations on each core. We can see two idle phases for each core, one when waiting to access the GPU and the other when waiting for the other cores to complete there computation.

**Figure 9:**
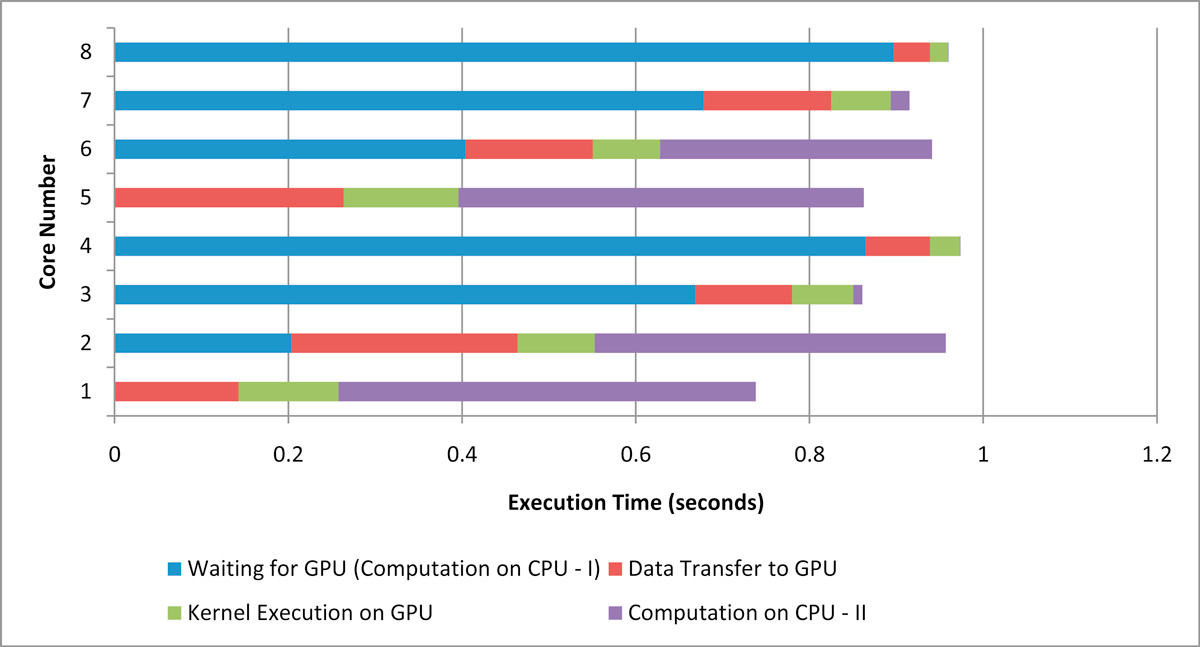
**Cooperative kernel offload profile**.

Figure 9 shows the execution profile of all 8 CPU cores while applying the cooperative offload method. We can see that the CPU and GPU cores are kept busy for a much larger percentage of time and finish at approximately, which indicates effective co-scheduling. Also, the EpiSimdemics application cannot be offloaded in exclusive mode, because it is inherently CPU-centric and task re-distribution is not possible. So, EpiSimdemics is offloaded in round-robin mode. EpiSimdemics is found to be 9.5-times faster (kernel speedup) than the single core CPU-only solution when the cooperative offload method is applied.

**Figure 10:**
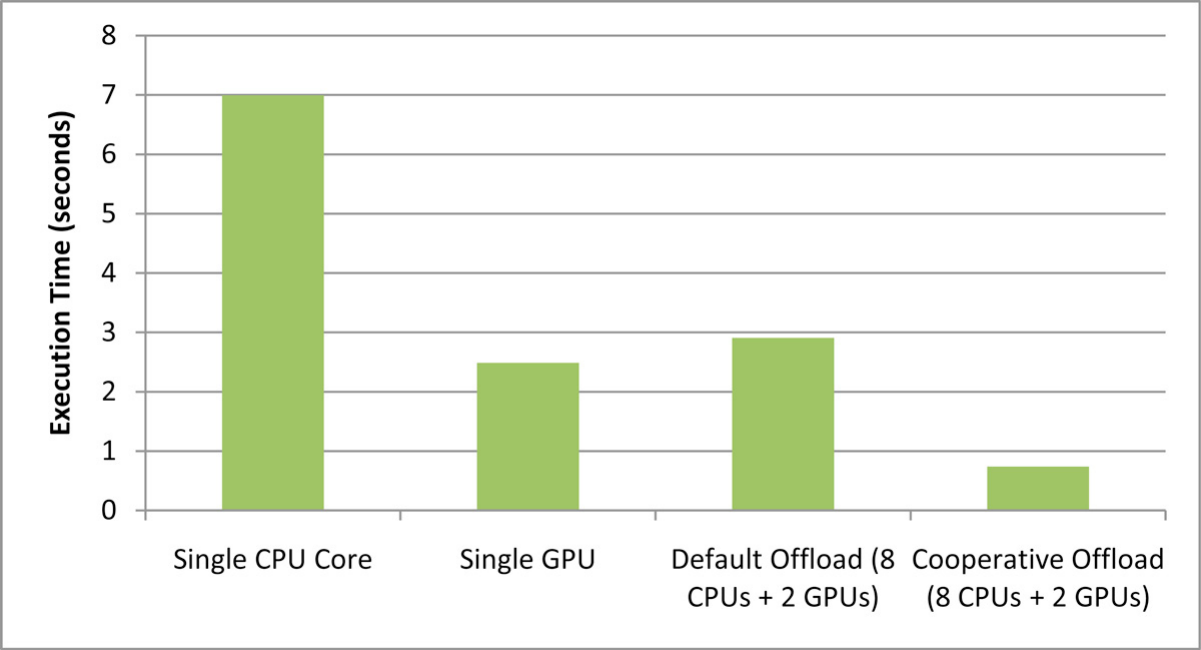
**Performance comparison of the different offload methods**.

Figure 10 shows the performance comparison (kernel speedup) of each of the offloading techniques with the CPU-only version of EpiSimdemics. We can see that the cooperative offload method shows the best performance.

### Future work

Interaction-based simulation systems can be used to model disparate and highly relevant problems in biology. We have shown that offloading some of the work in distributed interaction-based simulations can be an effective way to achieve increased intra-node efficiency. When combined with effective techniques for internode communication, high scalability can be achieved. We also described ways to exploit multiple cores and multiple GPUs per node by intelligently scheduling work across all available processors. In the future, we plan to explore using other GPU frameworks and GPU-aware communication libraries that reduce the data transfer overhead between the CPU memory and the device memory of the GPU.

We are currently developing a model of gut immunity. The goals of this model are to build, refine and validate predictive multiscale models of the gut mucosal immune system that compute condition-based interactions between cells for understanding the mechanisms of action underlying immunity to enteric pathogens. Instead of people moving among locations as in the Computational Epidemiology model, immune cells move among tissue locations. Individual immune cells make contact and interact with dynamic populations of bacteria and cytokines as they migrate within and among three tissue sites: i) the lumen/lamina propria border, where epithelial cells reside, ii) the lamina propria, more generally termed the effector site of the immune response, and iii) mesenteric lymph node, the inductive site of the immune response. This level of detail requires unprecedented scaling on high-performance computing systems - 10^7 ^to 10^9 ^cells (agents), simulations with time resolution of minutes for a total period of years and spatial resolution of 10^−4 ^meters.

## Competing interests

The authors declare that they have no competing interests.

## Authors' contributions

KB conceived the project, designed and implemented EpiSimdemics and its mathematical models, helped in the design of EpiSimdemics on the GPU, and drafted the manuscript. AA studied the GPU architecture, designed and implemented EpiSimdemics on the GPU and drafted the respective portions of the manuscript. MM designed the mathematical models and the original EpiSimdemics algorithm. WF conceived the GPU project and participated in its design and coordination and helped to draft the manuscript. All authors read and approved the final manuscript.
